# Hydrogen peroxide can be a plausible biomarker in cyanobacterial bloom treatment

**DOI:** 10.1038/s41598-021-02978-6

**Published:** 2022-01-07

**Authors:** Takashi Asaeda, Mizanur Rahman, Helayaye Damitha Lakmali Abeynayaka

**Affiliations:** 1 Saitama University, Saitama, 338-8570 Japan; 2 Hydro Technology Institute, Shimo-meguro, Tokyo, Japan; 3 Research and Development Center, Nippon Koei, Tsukuba, Japan

**Keywords:** Environmental impact, Ecology, Environmental sciences

## Abstract

The effect of combined stresses, photoinhibition, and nutrient depletion on the oxidative stress of cyanobacteria was measured in laboratory experiments to develop the biomass prediction model. *Phormidium ambiguum* was exposed to various photosynthetically active radiation (PAR) intensities and phosphorous (P) concentrations with fixed nitrogen concentrations. The samples were subjected to stress assays by detecting the hydrogen peroxide (H_2_O_2_) concentration and antioxidant activities of catalase (CAT) and superoxide dismutase (SOD). H_2_O_2_ concentrations decreased to 30 µmol m^−2^ s^−1^ of PAR, then increased with higher PAR intensities. Regarding P concentrations, H_2_O_2_ concentrations (nmol L^−1^) generally decreased with increasing P concentrations. SOD and CAT activities were proportionate to the H_2_O_2_ protein^−1^. No H_2_O_2_ concentrations detected outside cells indicated the biological production of H_2_O_2_, and the accumulated H_2_O_2_ concentration inside cells was parameterized with H_2_O_2_ concentration protein^−1^. With over 30 µmol m^−2^ s^−1^ of PAR, H_2_O_2_ concentration protein^−1^ had a similar increasing trend with PAR intensity, independently of P concentration. Meanwhile, with increasing P concentration, H_2_O_2_ protein^−1^ decreased in a similar pattern regardless of PAR intensity. Protein content decreased with gradually increasing H_2_O_2_ up to 4 nmol H_2_O_2_ mg^−1^ protein, which provides a threshold to restrict the growth of cyanobacteria. With these results, an empirical formula—protein (mg L^−1^) = − 192*Log((H_2_O_2_/protein)/4.1), where H_2_O_2_/protein (nmol mg^−1^) = − 0.312*PAR^2^/(50^2^ + PAR^2^)*((25/PAR)^4^ + 1)*Log(P/133,100), as a function of total phosphorus concentration, P (µg L^−1^)—was developed to obtain the cyanobacteria biomass.

## Introduction

Cyanobacteria blooms often produce toxic metabolites and are harmful to other organisms as well as humans. Hydrogen peroxide (H_2_O_2_) is often endorsed to reduce cyanobacterial abundance and organic pollutants as it is more effective in application with cyanobacteria compared to other phytoplankton^1,2^. Various experimental approaches have been completed in wastewater treatment, such as advanced oxidation processes, investigation of the degradation of aniline, and evaluation of kenaf fibers and water reservoirs by biological methods^3–6^. However, H_2_O_2_ is also produced by other factors. First, H_2_O_2_ is generated photochemically from dissolved chromophoric organic materials exposed to UV, and H_2_O_2_ distribution was observed in natural lakes^7–9^. At the same time, H_2_O_2_ is biologically produced in cells exposed to environmental stresses, including metal ion toxicity, salinization, temperature, PAR conditions, eutrophication, allelopathy, and pathogens. However, its contribution to the total concentration is unknown.

Under a stress environment, endogenous reactive oxygen species (ROS) production, including superoxide, hydroxyl radicals, and H_2_O_2_ concentration exceeds its scavenging capacity^10–12^. ROS are essential for growth regulation and signaling mechanisms in photosynthetic organisms^13,14^. Those organisms, in turn, are capable of controlling excess ROS production with their inherent scavenging enzymes and non-enzymatic components^13,15^. Accumulation of excessive ROS inside cells causes harmful impacts on cyanobacteria, such as disrupting cellular homeostasis, causing membrane lipid peroxidation, protein oxidation, enzyme inhibition, and DNA and RNA damages. It also affects the photosynthetic apparatus, leading to cell mortality as the concentration exceeds the threshold value^16^.

Cyanobacteria are sensitive to even a minor change in light intensity as they usually expose relatively weak light; thus, even moderate solar radiation may cause stress^17^. The collection of solar energy at photosystem II (PSII) in the thylakoid membrane results in the oxidation of water molecules and reduction of plastoquinone, a molecule involved in the electron transport chain. The produced electrons are transported to PSI, where they are consumed in the synthesis of carbohydrates. However, an overabundance of solar energy results in the generation of ROS, including superoxide radicals, as the energy transfer rate is limited due to the underutilization of energy absorbed by the PSII antenna complex in the PSII reaction center^15,18–20^. Superoxide dismutase (SOD) catalyzes superoxide radicals into H_2_O_2_ before being detoxified into water by antioxidant activities^21^. However, the high oxidation potential of ROS can lead to the destruction of proteins, which otherwise recover the photosystem activities^22^. Thus, excessive solar radiation inhibits the proliferation of cyanobacteria. Similarly, the shortage of nutrient conditions, including P and nitrogen (N), is identified as a dominant stressor that suppresses the growth of cyanobacteria^23^. P is an important macronutrient to plankton growth in many ways. It makes rigid structures in cell walls, membranes, and nucleic acids by making covalent links between monomers. It is also involved in cell metabolism directly by storing energy as polyphosphate bodies in plankton cells^24^. The absolute concentrations of P and N and the stoichiometric ratio of these elements often play an important role in plankton growth in lakes^25^. N:P mass ratio varies between 240 and 0.5, depending on the variation of P concentration in lakes^26^. When the mass ratio of N:P exceeds 10, P is considered as the limiting factor. On the other hand, when N:P less than 10, N becomes the limiting factor on phytoplankton growth, including cyanobacteria in freshwater bodies^27^. Hence, both surplus and deficiency of nutrients could cause significant alternations in cyanobacteria biomass and cellular stress. The combined effect of various abiotic stresses on the production rate is often reported^28,29^. Some combinations inhibit growth due to the contradicting impacts of stressors; however, a significant reduction of biomass is also reported as caused by simultaneous exposure to multiple stressors compared to a single stress source^30^. Hence, excessive radiation stress combined with a shortage of P and N nutrients could generate huge cellular stress and cyanobacterial growth inhibition.

The concentration of H_2_O_2_ and the activity of antioxidant enzymes are some of the biomarkers employed in stress detections. The role of H_2_O_2_ in plants and how they respond to environmental stress has been a focus throughout the literature^31–33^, suggesting a potential to develop ROS-based strategies for predicting cyanobacterial bloom formation and H_2_O_2_ concentrations^34^. Thus, this research was designed to study the (1) effects of the PAR regime and P concentrations on cyanobacteria stress, particularly endogenous H_2_O_2_ concentrations, under the condition of naturally produced H_2_O_2_ from organic matter, (2) combined effects of the PAR regime and P concentrations on H_2_O_2_ concentrations, and (3) relationship between H_2_O_2_ concentrations and antioxidant enzyme activities of cyanobacteria, aiming at the possibility of applying H_2_O_2_ concentrations as a proxy to detect stress intensity in algal management and the contribution rate of the biological H_2_O_2_ production rate in the treatment.

## Results

### The effect of PAR intensity and phosphorous concentration on H_2_O_2_ concentration

**Figure 1:**
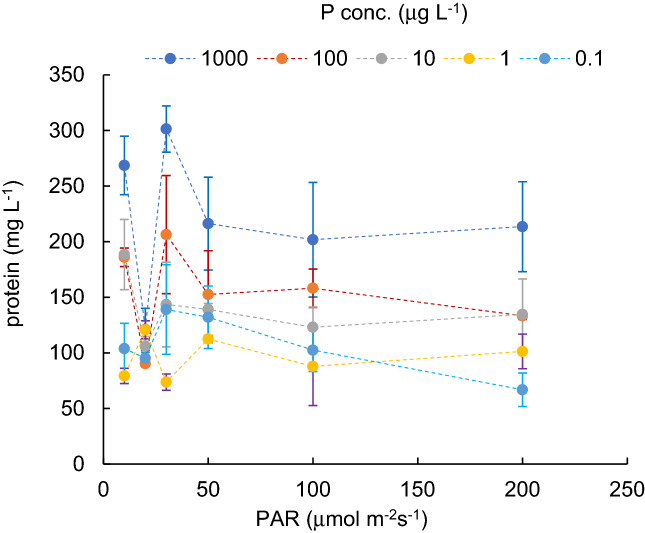
Protein content for different PAR intensity levels and for each phosphorus concentration level. Vertical bars indicate standard deviation.

**Figure 2:**
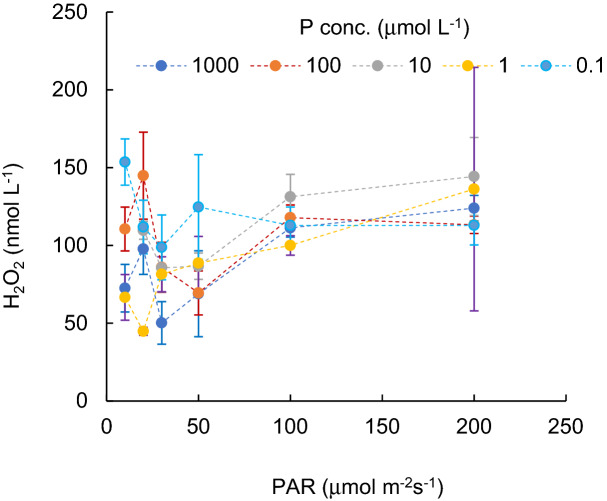
H_2_O_2_ concentration for different PAR intensity levels and for each phosphorus concentration level. Vertical bars indicate standard deviation.

**Figure 3:**
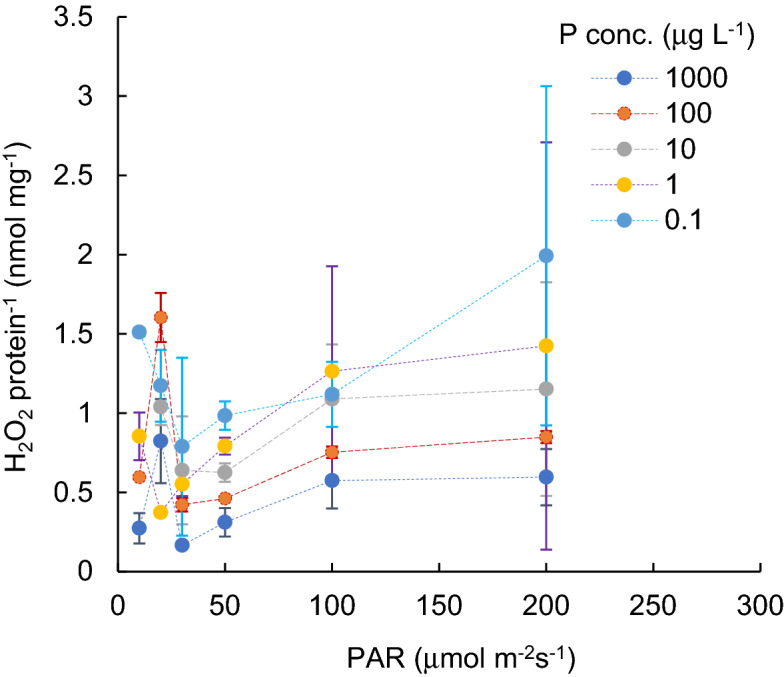
H_2_O_2_ content per protein for different PAR intensity levels and for each P concentration level . Vertical bars indicate standard deviation.

**Figure 4:**
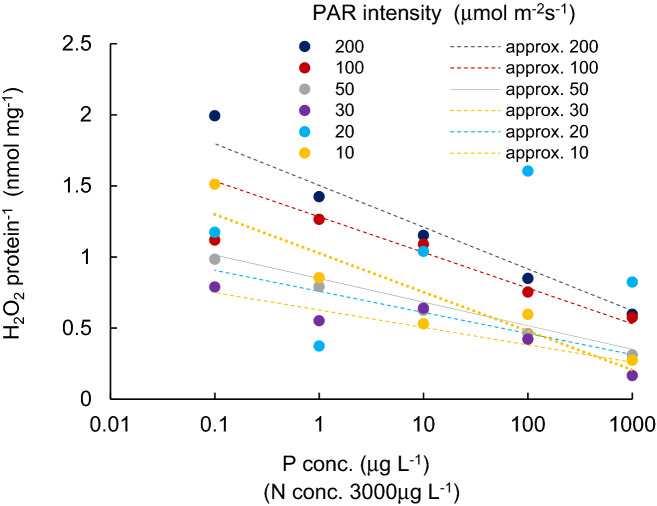
H_2_O_2_ content per protein for different P concentration level and for each PAR intensity level. Vertical bars indicate standard deviation. Dotted lines show the approximate relation for each light intensity.

Variations in protein contents of cyanobacterial cultures (mg L^-1^), grown under different PAR intensity levels with different P concentrations, are shown in Fig. 1. Figure 2 indicates the H_2_O_2_ concentration variations (nmol L^-1^) for different PAR intensity levels and each P concentration level. Vertical bars indicate standard deviation. Higher protein content was obtained when PAR exposure was lower than 50 µmol m^−2^ s^−1^. In a PAR intensity range between 0 and 30 µmol m^−2^ s^−1^, the H_2_O_2_ concentration declined from 50 to 150 nmol L^−1^ at dark condition, with increasing PAR. With a PAR intensity between a 30 and 200 µmol m^−2^ s^−1^ range, the protein content was slightly reduced (R^2^ = − 0.06, *p* > 0.1), while the H_2_O_2_ concentration significantly increased (R^2^ = 0.73, *p* < 0.001, and 0.910, 0.720, 0.92, 0.92 and 0.16 for 1000, 100, 10, 1.0, 0.1 μg L^−1^ of P concentration, respectively), regardless of the P concentration. The variational trend of H_2_O_2_ concentration per protein (nmol mg^-1^) is shown in Fig. 3 with respect to the PAR intensity. Regardless of the P concentration, it declined with low PAR intensities to 30 µmol m^−2^ s^−1^ of PAR intensity and then increased with a decreasing enhancement rate. At values higher than 50 µmol m^−2^ s^−1^ PAR, no significant difference was obtained in the variational trend of the H_2_O_2_ per protein with respect to PAR. The increasing trend of H_2_O_2_ per protein with PAR intensity is mainly attributed to the increasing trend of H_2_O_2_ concentration rather than the reduction of protein content. H_2_O_2_ per protein was generally lower with a higher P concentration (*p* < 0.03). H_2_O_2_ per protein, measured as high as 0.2 up to 2.0 nmol mg^−1^ with 10–200 µmol m^−2^ s^−1^of PAR intensities, declined with a higher P concentration uniquely except for 20 μmol m^-2^ s^-1^ (Fig. 4).

### Antioxidant activities with respect to H_2_O_2_ concentration per protein

**Figure 5:**
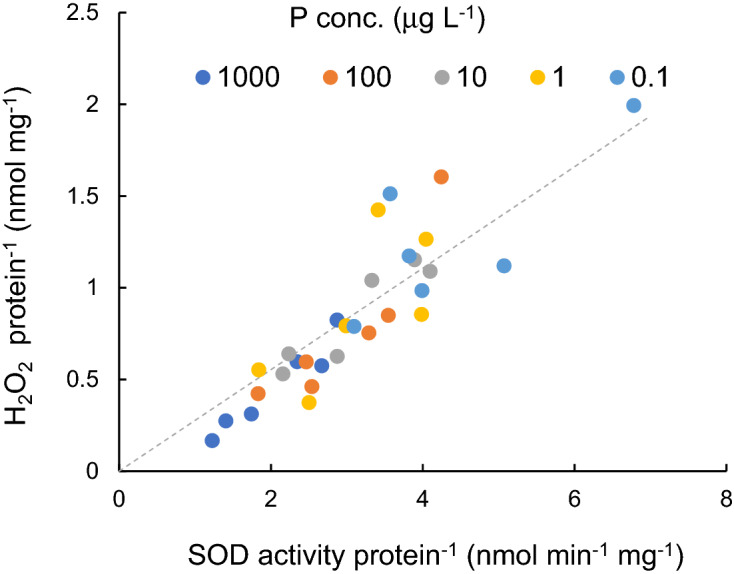
Relation between SOD activity and H_2_O_2_ concentration for different P concentration level. The approximate relation is shown by the diagonal line.

SOD activity per protein was uniquely proportionate to H_2_O_2_ per protein (Fig. 5). The approximate relation is shown by the diagonal line, where the H_2_O_2_ protein^-^^1^ (nmol mg^−1^) = 0.276(min)*SOD (nmol mg^−1^ min^−1^), (R^2^ = 0.950, *p* < 0.01).

**Figure 6:**
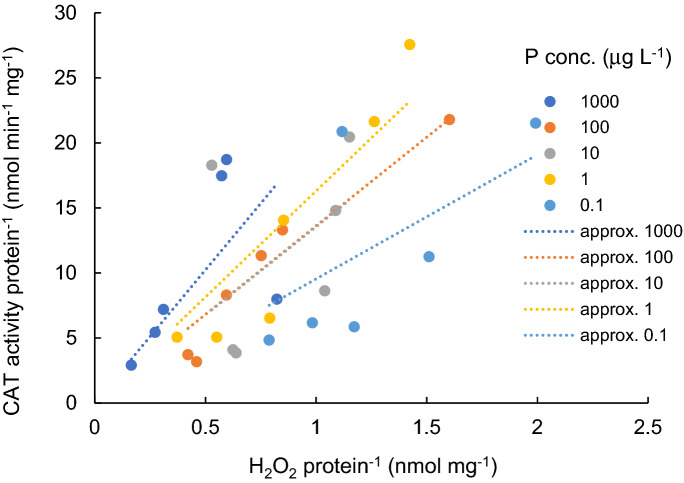
Relation between H_2_O_2_ concentration and CAT activity for different P concentration levels. Dotted lines indicate the approximate lines for each P concentration.

CAT activity per protein is shown as a function of H_2_O_2_ concentration per protein, separately shown by each P concentration in Fig. 6. For each P concentration level, CAT activity per protein linearly increased with the H_2_O_2_ protein^-^^1^. The increasing rate was higher based on PAR intensity (18.73 CAT H_2_O_2_^-^^1^, R^2^ = 0.573 for 1000 µg L^−1^, 13.82 CAT H_2_O_2_^-1^, R^2^ = 0.977 for 100 µg L^−1^; 12.89 CAT H_2_O_2_^-1^, R^2^ = 0.793 for 10 µg L^−1^; 14.53 CAT H_2_O_2_^-1^, R^2^ = 0.949 for 1 µg L^−1^; and 9.22 CAT H_2_O_2_^-1^, R^2^ = 0.766, for 0.1 µg L^−1^), and the proportional coefficient was found to have a significant positive correlation with the logarithmic scale of the P concentration (*p* < 0.01).

On the other hand, for each PAR intensity level, CAT activity did not have significant positive correlation with the P concentration level.

## Discussion

### The effect of biologically produced H_2_O_2_ on the suppression of cyanobacterial blooms

The artificial endorsement of H_2_O_2_ has a high potential to suppress cyanobacterial blooms with less effect on other organisms compared to other controlling methods^35–39^. Previous researchers obtained the lethal H_2_O_2_ dosage for cyanobacteria by laboratory incubations under different H_2_O_2_ concentrations; cyanobacterial chlorophyll declined to nearly half after an 18 h incubation with approximately 30 µmol L^−1^ of H_2_O_2_^1,40^ or after a 4 h incubation with 100 µmol L^−1^ of H_2_O_2_^2^ H_2_O_2_ delayed fluorescence decay with 0.1 µmol of H_2_O_2_ L^−1^^7^. At the same time, the Fv/Fm value substantially declined with 100 µmol of H_2_O_2_ L^−1^^41^, and dead cells increased with 275 µmol of H_2_O_2_ L^−1^^39^. Cyanobacteria were in a lethal condition^1,2^ and sub-lethal at concentrations exceeding 50 µmol of H_2_O_2_ L^−1^^39^. All previous experimental results reveal that cyanobacterial biomass is degraded with higher H_2_O_2_ concentrations; however, the H_2_O_2_ concentration threshold varies widely from 1 to 1000 µmol L^−1^. In the present study, we used protein content as an indicator of biomass rather than the chlorophyll content of *Phormidium ambiguum* cells because chlorophyll-a can be expressed on a protein basis^42^. However, the decreasing trend of protein content, which was seen with the Chl-a concentration, was also observed with H_2_O_2_ concentrations.

Natural H_2_O_2_ formation has been identified in aquatic ecosystems as photolysis of dissolved organic carbon (DOC) exposed to UV^7,43,44^. Then it is reported that the H_2_O_2_ production varies with the nutrient content of the water body. However, the H_2_O_2_ concentration of these waters was in the magnitude of µmol L^−1^^8,34,45^. The comparison of these results indicates that the photolysis of organic carbon in natural water only is not sufficient to control cyanobacterial biomass.

H_2_O_2_ is also produced biologically and is accumulated in cells subject to high levels of environmental stress. In the present study, UV was limited. Accordingly, measured H_2_O_2_ was considered a biologically produced component in cells or cell surfaces, which was then released into the ambient water. In the present experiment, protein content was measured as a reference of the biomass of cyanobacteria. Cell biomass is two to three times larger with protein content^46^.

As the buoyancy of the cells is nearly neutral, the H_2_O_2_ content per protein, ~ 1 mmol of H_2_O_2_ kg^−1^, was generated and contained in the cell before release. This constitutes more or less the same level of the lethal H_2_O_2_ concentration in water.

**Figure 7:**
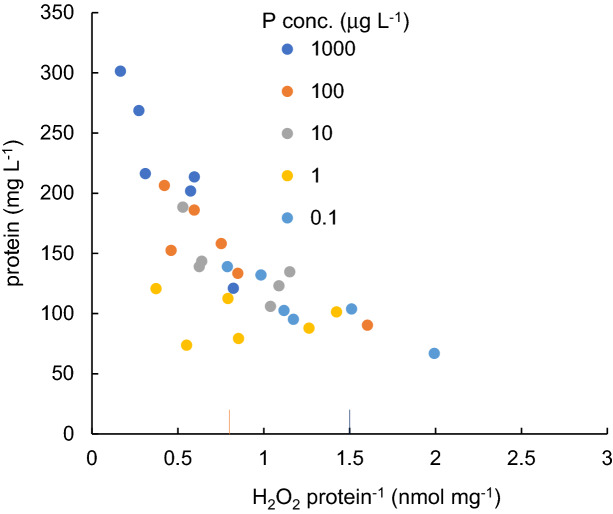
Relation between H_2_O_2_ concentration per protein and protein content in waterfor different P concentration levels. Lethal level of cyanobacteria in the previous reports^35,39^ are shown for comparison.

The protein content in water declined with an increasing H_2_O_2_ concentration per protein up to 2 nmol mg^−1^ protein (Fig. 7). It was nearly same as the lethal level of the previous studies^35,39^. A higher protein level was not observed with higher H_2_O_2_ concentration levels in the present study. The growth of cyanobacteria is suppressed by the generation of higher H_2_O_2_ levels.

The lethal H_2_O_2_ concentration obtained here corresponds well with about 16 µmol of H_2_O_2_ g^−1^ FW of a threshold condition to grow *Egeria densa* in natural water^33^, considering the weakness of cyanobacteria to H_2_O_2_ rather than other plant species^35^.

### The possible indicator of environmental stress and the effect of combined stress factors

The accumulation of ROS is reported to augment in parallel fashion to increased abiotic stress^47–49^. In the present experiment, two types of abiotic stresses, phosphorous deficiency and high or low PAR intensities, were applied with different intensities of each.

Though H_2_O_2_ is produced under normal environmental conditions, its production is accelerated under high stress intensity. In natural water, cyanobacteria often suffer from a shortage of N and P. Stoichiometrically, the ratio of the N and P of cyanobacterial cells is approximately 16:1^50^. Waters with an N:P ratio of < 15 are most susceptible to cyanobacterial dominance^51,52^. In the present experiment, the P concentration was changed with the fixed amount of an N concentration of 3000 µg L^−1^. Thus, the P concentration becomes restrictive, except for 1000 µg L^−1^ in the present study’s conditions. A significant increasing trend was observed in H_2_O_2_ per protein with a decreasing P concentration. The deficiency of essential nutrients may increase oxidative stress and then deteriorate the growth rate of cyanobacteria.

Under all tested P concentrations, H_2_O_2_ per protein content decreased with increasing PAR intensity until 30 µmol m^−2^ s^−1^, taking the lowest value there, then grew at higher PAR intensities, though the increasing rate gradually decreased. The enhanced production of H_2_O_2_ under prolonged low PAR conditions has not been sufficiently studied, though superoxide production in dark conditions is reported^26^. With submerged macrophytes, *Egeria densa*, the H_2_O_2_ concentration was the lowest empirically under the prolonged exposure of a PAR intensity level of approximately 60 µmol m^−2^ s^−1^^33,53^. The H_2_O_2_ concentration increased both with decreasing or increasing PAR intensities. However, the underlying mechanisms are unknown.

The increasing H_2_O_2_ concentration per protein over 30 µmol m^−2^ s^−1^ of PAR intensity is attributed to the excessive harvesting of PAR energy^17,18^. In the thylakoid membrane, electrons are produced by solar energy and transmitted to plastoquinone in PSII, which are partially accepted for carbon dioxide fixation. More electrons are generated when exposed to higher levels of solar radiation. Consequently, the photoinhibition of photosystem-II (PS-II) is induced, leading to oxidative damage because of the generated ROS such as superoxide, hydroxyl radicals, and H_2_O_2_. It damages cellular components, such as the D1 protein, which otherwise mends the damaged photosynthesis apparatus^18^.

The process comprises the direct reduction of O_2_ by PS-I, resulting in singlet oxygen production followed by superoxide, which is converted to H_2_O_2_ by the activities of the enzyme SOD. In the present study, H_2_O_2_ per protein was proportionate with SOD activity, generating H_2_O_2_ from superoxide. CAT activity was far higher than other major antioxidant activities to decompose H_2_O_2_ and linearly increased with H_2_O_2_ concentration.

Though SOD and CAT activities demonstrated different dependencies on PAR intensity levels and the P concentration, their activities were evaluated by the single function with H_2_O_2_ per protein. A steady H_2_O_2_ concentration is sustained by balancing the generated H_2_O_2_ with different types of stresses and these antioxidant activities as a single function of H_2_O_2_ content per protein.

In natural water, cyanobacteria are exposed to various abiotic stresses that enhance oxidative stress, producing H_2_O_2_, which may deteriorate cyanobacterial biomass. A significant negative correlation was recognized for protein content with respect to the H_2_O_2_ concentration (n = 90, R^2^ = − 0.712, *p* < 0.01), irrespective of stress types.

The production rate of H_2_O_2_ is not necessarily cumulative for different types of abiotic stresses^28,30^. However, the H_2_O_2_ concentration was enhanced with increasing PAR intensity and decreasing phosphorus concentration, respectively, and the enhancement of the H_2_O_2_ concentration was independent of each other (*p* < 0.01). The total H_2_O_2_ per protein is empirically given as the sum of H_2_O_2_ produced by the intensity of each stress component at least as a practical use level. Thus, the total H_2_O_2_ concentration is approximately provided by the sum of the H_2_O_2_ concentration attributed to each stress. The same trend was obtained for submerged macrophytes^33,53^. Consequently, a potential for using the H_2_O_2_ concentration to estimate cyanobacterial biomass exists.

### The estimation of H_2_O_2_ concentrations produced by cyanobacteria under abiotic stresses

For the application of the empirically obtained results to practical use in the prediction of algal blooms in the environment where PAR and P concentrations are restricted factors for growth, the trend of the H_2_O_2_ per protein (nmol mg^−1^) is obtained as a function of PAR (µmol m^−2^ s^−1^) and the P concentration, P (µg L^−1^), as formulated by:

1 $${\text{H}}_{2} {\text{O}}_{2} {\text{/protein}} = - 0.312*{\text{PAR}}^{2} {/}\left( {50^{2} + {\text{PAR}}^{2} } \right)*\left( {\left( {25{\text{/PAR}}} \right)^{4} + 1} \right)*{\mathrm{Log}}\left( {{\text{P/}}133{,}100} \right)$$

where 0.1 µg PL^−1^ < P < 1000 µg PL^−1^, 30 µmol m^−2^ s^−1^ < PAR, and protein represents the amount of protein in mg L^−1^.

**Figure 8:**
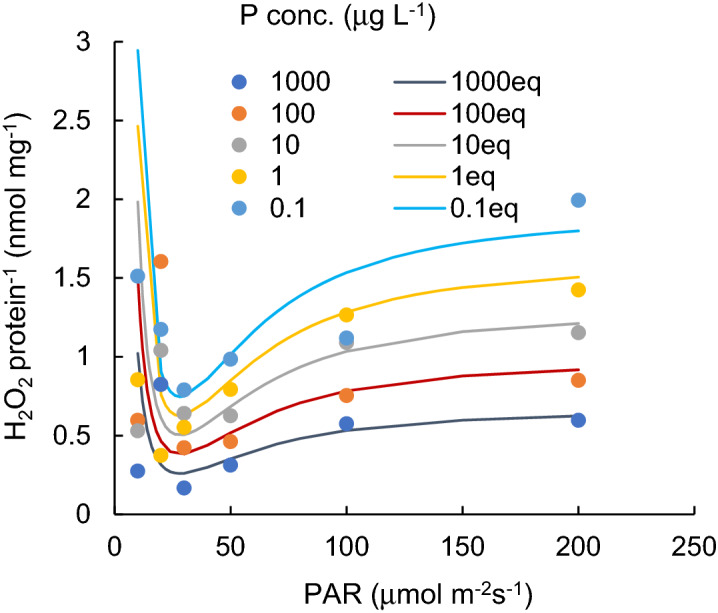
The simulated results of H_2_O_2_/protein by Eq. (1) compared with experimental results.

The relationship is shown in Fig. 8. The simulated results of the H_2_O_2_ protein^−1^ by Eq. (1) compared with experimental results and a significant similarity was obtained (R^2^ = 0.953, *p* = 0.012, for 1000 µg L^−1^, R^2^ = 0.696, *p* = 0.0065 for 100 µg L^−1^; R^2^ = 0.927, *p* = 0.023 for 10 µg L^−1^; R^2^ = 0.982, *p* = 0.00289 for 1 µg L^−1^; R^2^ = 0.024, and *p* = 0.024 for 0.1 µg L^−1^).

**Figure 9:**
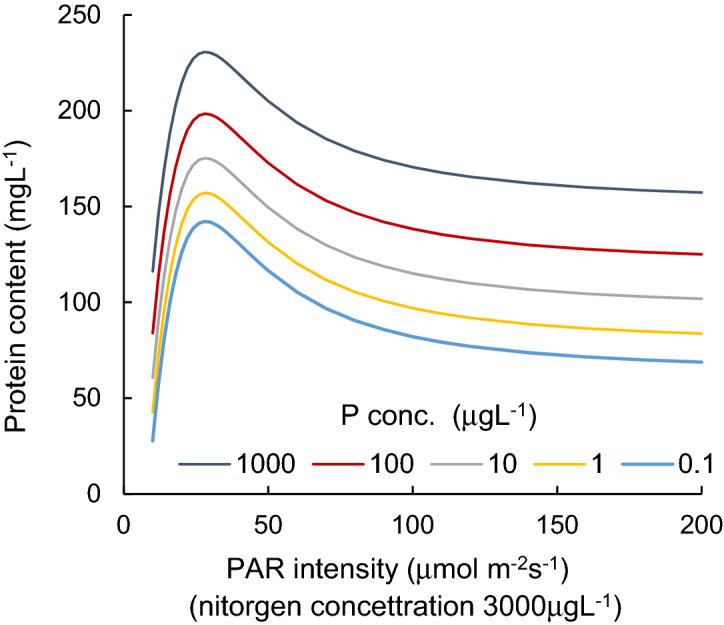
The simulated results of protein content.

The protein content (mg L^−1^) is shown in Fig. 9. The simulated results of protein content by Eq. (2), as a function of H_2_O_2_ per protein, was shown to possess significant negative correlation (R^2^ = − 0.675, *p* < 0.01), which is empirically formulated by:

2 $${\text{protein}} = - 192*{\text{Log}}(({\text{H}}_{2} {\text{O}}_{2} {\text{/protein}}){/}4.1)$$

(R^2^ = − 0.71, *p* < 0.01).

With Eqs. (1) and (2), protein content is estimated as a function of PAR and the P concentration.

The estimated protein contents are denoted in Fig. 9. Protein content in water for different P concentration levels (mg L^−1^) and for each PAR intensity level (µmol m^−2^ s^−1^). The concentration uniquely increased with increasing P concentrations.

The cellular growth rate gradually decreased with light intensity^54^, and the growth rate of cyanobacteria reached a maximum at 30–50 µmol m^−2^ s^−1^^55 ^. *P. ambiguum* prefers relatively low light intensity at ~ 18 µmol m^−2^ s^−1^^56  ^. The diagram seems to provide reasonable results.

## Conclusions

The endogenous H_2_O_2_ concentration is an effective tool to detect the stress level of cyanobacteria. Both PAR regimes and P concentration shortages are shown to enhance H_2_O_2_ concentrations in cyanobacterial cultures.

H_2_O_2_ per protein content declines in low PAR conditions and increases when exposed to higher PAR intensity levels while generally increasing as the P concentration decreases. H_2_O_2_ per protein for combined stresses is given by the sum of the amount produced by each stress. Protein content decreases uniquely following the value of H_2_O_2_ per protein in a cyanobacterial culture. Then the H_2_O_2_ per protein exceeds the threshold value, cyanobacteria will decline. As a result, cyanobacterial cell will lyse leading to death. Cyanobacterial cell biomass can easily be regulated by this way.

The prediction model was developed for the protein content to design management criteria for excessive cyanobacterial blooms in freshwaters.

### Methodology

#### Culture and incubation

*Phormidium ambiguum*, an odor-forming benthic cyanobacterial species, was obtained from the National Institute of Studies (NIES), Japan. The strain was cultured and acclimatized for 30 days in an autoclaved BG 11 medium^57^, maintained at 20 °C under controlled PAR conditions with white fluorescent light, having flux of 20 µmol m^−2^ s^−1^ in a light-and-dark cycle of 12 h:12 h. The cultures were manually shaken twice a day. Cells were subcultured by diluting with new BG 11 medium as needed.

### Experimental procedure

After 30 days, well-grown cyanobacterial cells were collected by centrifugation, washed once with distilled water, and then re-suspended in modified BG 11 media. All experiments were conducted by using incubators (MIR-254, Sanyo, Tokyo, Japan) with a nutrient level of BG-11 medium consisting of NaNO_3_ 17.6 mM, K_2_HPO_4_ 0.2296 mM, MgSO_4_.7H_2_O 0.0146 µM, Na2CO3 0.0189 µM, Citric acid 0.0031 µM, Ferric ammonium citrate 0.0023 µM, EDTA (Na_2_ salt) 0.0297 µM, H_3_BO_3_ 4.6253 μM, MnCl_2._4H_2_O 0.9145 µM, ZnSO_4_.7H2O 0.0765 µM, Na_2_MoO_4_.2H_2_O 0.1611 µM, CuSO_4_.5H_2_O 0.0316 µM, and Co(NO_3_)_2_.6H_2_O 0.0023 µM in 1 L of deionized water^58^, adjusted for N and P concentrations, respectively, at 3000 µg L^−1^ of nitrogen and 0.1, 1.0, 10, 100, and 1000 µg L^−1^ of P. Six different PAR intensities—namely, 10, 20, 30, 50, 100 and 200 µmol m^−2^ s^−1^—by white fluorescent light (Toshiba, Japan) and VBP-L24-C2 PAR source (Valore, Kyoto, Japan) were used with 12 h:12 h PAR and dark cycle. The PAR intensities were measured using a quantum sensor (EKO Instruments Co., Ltd., Japan) and adjusted uniformly in the media. The temperature was kept constant (20 °C) throughout the experiment. At 12:00, after 7 days, samples were taken for the subsequent stress response analysis. Collected samples were subjected to bioassays that are described later.

### Analyses

#### Total soluble protein content analysis

Total soluble protein concentration was determined using the same method mentioned in^57^ with minor modifications. Cyanobacterial cells were extracted from 1 mL of culture media by centrifugation at 4 °C for 10 min at 10,000 rpm, and the pellet was washed once with distilled water. Then, the cell pellet was subjected to a freeze–thaw cycle. Total soluble protein was extracted using a 0.5 M NaOH solution, and the extraction was centrifuged at 4 °C for 20 min at 10,000 rpm. The supernatant was used as crude protein extract, and the protein content was quantitatively analyzed with the aid of a Coomassie Bradford protein assay kit. Crude protein extract was stained with Coomassie (G-250) dye and incubated for 10 min at room temperature, and then the absorbance was measured at 595 nm using a UV–Vis spectrometer (Shimazu, Japan). Protein content was determined using a known concentration series of Albumin.

### Stress assay

#### H_2_O_2_ assay

Cellular H_2_O_2_ contents were estimated according to the titanium chloride method^ 59^. A total of 750 μL of 0.1% titanium chloride in 20% H_2_SO_4_ (v/v) was then added to initiate the reaction. The optical absorption after 1 min was measured at 410 nm using a spectrophotometer (UVmini-1240). However, the absorption at 410 nm includes the effect of other soluble compounds^60–62^. Thus, the H_2_O_2_ concentration was calculated from the slopes of the standard curve obtained from known H_2_O_2_ concentrations, which was offset derived by the intercept absorption rate with zero H_2_O_2_ concentration samples^61^. The results were compared with those of the e-FOX method and a suitable agreement was obtained^62^.

#### CAT assay

The CAT activity was measured by reacting 15 µL of 750 mM H_2_O_2_, 920 µL of potassium phosphate buffer, and 65 µL of extract supernatant. Optical absorption was measured at 240 nm using UV mini-1240. The measurements were recorded every 20 s for 3 min, and CAT activity was calculated using an extinction coefficient of 39.4 mM^−1^ cm^ 63^. The scavenging rate of H_2_O_2_ by enzyme extract per minute was defined as unit of CAT activity per mg of total soluble protein.

#### APX assay

For the APX assay, the reaction mixture contained 100 µL of enzyme extract, 200 µL of 0.5 mM ascorbic acid in 50 mM potassium phosphate buffer (pH 7.0), and 2 mL of 50 mM potassium phosphate buffer (pH 7.0) mixed with 60 µL of 1 mM H_2_O_2_. The decrease in absorbance at 290 nm was recorded every 20 s for 3 min. The APX activity was calculated using an extinction coefficient of 2.8 mM^−1^ cm^−1^^64^.

#### SOD assay

Total SOD activity was determined by using methods as described by^65^. The reaction mixture contained 50 mM phosphate buffer (pH 7.8), 0.66 mM EDTA, 10 mM methionine, 33 µM NBT, 0.0033 mM riboflavin and 50 µL cyanobacterial enzyme extract. The reaction was allowed to proceed under fluorescent illumination. After that, the absorbance of the reaction mixture was read at 560 nm. One unit of SOD activity was defined as the number of enzymes required to cause 50% inhibition of the NBT photo-reduction. The results were expressed as unit of SOD activity per m﻿g of total soluble protein.

### Statistics

Variance (ANOVA) and the bivariate analysis were used and Pearson’s correlation method was followed to evaluate the relationship among parameters. Statistical analyses were performed with the help of IBM SPSS V25.

H_2_O_2_ concentrations showed correlations with increasing PAR intensities and decreasing P concentrations independently. The fitted curve patterns were different for each PAR intensity or each P concentration.

Therefore, the most fitted curves of H_2_O_2_ concentration with respect to P concentration, and with respect to PAR intensity, 30–200 µmol m^−2^ s^−1^, were obtained for each PAR intensity, and the P concentration, 0.1–1000 mg L^−1^, respectively. Then, the effect of PAR intensity and P concentration on the H_2_O_2_ concentration was estimated.

The variance (ANOVA) and the bivariate analysis were used and Pearson’s correlation method was followed to evaluate the relationship among parameters. Statistical analyses were performed with the help of IBM SPSS V25.
